# Association of Vitamin D Status with COVID-19 Infection and Mortality in the Asia Pacific region: A Cross-Sectional Study

**DOI:** 10.1007/s12291-020-00950-1

**Published:** 2021-02-03

**Authors:** Dharmveer Yadav, Amandeep Birdi, Sojit Tomo, Jaykaran Charan, Pankaj Bhardwaj, Praveen Sharma

**Affiliations:** 1 Department of Biochemistry, All India Institute of Medical Sciences, Jodhpur, Rajasthan India; 2 Department of Pharmacology, All India Institute of Medical Sciences, Jodhpur, Rajasthan India; 3 Department of Community and Family Medicine, All India Institute of Medical Sciences, Jodhpur, Rajasthan India

**Keywords:** COVID-19, Vitamin D levels, ACE-2, Asia pacific region

## Abstract

COVID-19 has been declared a global pandemic by WHO on 11 March 2020. Still, very little is known about the potential protective dietary factors for the prevention of infection and mortality due to COVID-19. Keeping in view the scarcity of literature/studies available, in this regards present study was undertaken to assess if there is any correlation between mean levels of Vitamin D in various Asia Pacific countries with the infection and mortality caused by COVID-19. We collected data for mean levels of Vitamin D for 37 Asia Pacific countries for which we have also got the data regarding the morbidity and mortality of COVID-19. The mean levels of Vitamin D were found to have a significant association with the number of cases/million(r =  − 0.394, *p* value = 0.016) and a weak association with the number of deaths/ million (r =  − 0.280, *p* value = 0.093) due to COVID-19. In conclusion, we found a significant relationship between Vitamin D levels with the number of COVID-19 cases. So further clinical trial/study with a large sample size is needed to elucidate the protective role of Vitamin D in COVID-19.

## Introduction

Vitamin D, a fat-soluble vitamin, regulates the absorption of calcium, phosphorous, and magnesium. Vitamin D also is known to play a vital role in increasing immunity against individual viral organisms and hence reducing mortality due to infection [1]. It helps in improving both natural and adaptive immunity [2]. Disruption of gap junction has been one of the mechanisms employed by viruses to gain entry into the target cell. Vitamin D prevents viral entry into the cell by maintain integrin proteins and helping in maintaining tight gap junctions [3–6].

Vitamin D is synthesized in the skin on exposure to sunlight. Seven hydroxycholesterol is converted to secosterol which subsequently gets converted to cholecalciferol. Cholecalciferol enters the liver and gets converted to 25 hydroxycholecalciferol. 25 hydroxycholecalceferol transported to the kidney, where it gets converted to the active form of vitamin D calcitriol [7]. Vitamin D enters the cell by binding to its receptor vitamin D receptor (VDR) and brings about its regulation of transcriptional activity of different target genes by binding to vitamin D response elements on DNA [7]. Vitamin D plays a role in innate immunity by producing peptides like cathelicidin and interleukin (IL) 37, which acts as an antimicrobial agent [8, 9]. Cathelicidins show its direct killing effect on enveloped and non-enveloped viruses as well as on some bacteria and fungi [10]. These peptides disturb cell membranes and neutralize endotoxins, leading to efficient clearance of invading pathogens [11]. Further, an animal study has shown that a decrease in IL-37 leads to increased replication of influenza A viruses [11].

Amidst the current scenario of the Covid- 19 pandemic, studies are being conducted to elucidate the role of vitamin D in the prevention of SARS- COV 2 virus infection. The entry of this virus into the cell requires angiotensin-converting enzyme 2 (ACE-2). This virus binds to the receptor and gains entry into the cell, and as it attaches to the receptor, it leads to a decrease in the expression of ACE 2. ACE2 receptors are present in epithelial cells of the lungs, blood vessels, intestine, kidney, and heart. ACE 2 functions by degrading angiotensin I to angiotensin I-IX and angiotensin 2 to angiotensin I-VII. Angiotensin I-IX binds to the *Mas* receptor and demonstrates anti-inflammatory and antioxidant and vasodilatory effects. It is stated that the binding of the SARS-COV 2 virus to the ACE receptor leads to a decrease in the anti-inflammatory response of ACE 2 and leads to the recruitment of inflammatory cytokines and leads to cytokine storm [12, 13].

The current study aimed to assess the correlation between Country-wise average Vitamin D levels and COVID-19 cases and mortality in countries in the Asia Pacific region. The study would provide insight into the protective role of Vitamin D in the infectivity and mortality of COVID-19.

## Methods

We performed an inclusive search of the literature for studies on vitamin D levels and COVID-19 from September 2000 up to 15th September 2020. All observational studies reporting the vitamin D levels from the Asia Pacific countries were searched using a different combination of MeSH terms related to the COVID-19 and vitamin D in Asia pacific countries through PubMed and google scholar. Asia Pacific countries were defined as per the Asia Pacific Observatory on Health Systems and Policies, WHO Regional Office for South–East Asia. Furthermore, references in the relevant articles were also searched to check for other eligible articles. Recent studies depicting average Vitamin D levels for countries in the Asia Pacific region were included for Vitamin D levels. Vitamin D levels mentioned in different units were standardized to ng/mL. Data for Vitamin D levels were obtained for 37 countries which were included in the analysis (Table 1) for few countries no data was available hence they were excluded. Articles were searched and reviewed for eligibility using the following inclusion criteria 1) mean Vitamin D levels in Asia pacific countries; 2) studies published in English.

**Table 1:** Vitamin D levels, COVID-19 cases and COVID-19 deaths per million in Asia Pacific countries

Sr. No	Country	Cases per million	Deaths per million	Vit D levels nmol/L
1	India	3565	58	24.3
2	Iran	4805	277	61.3
3	Bangladesh	2056	29	54.2
4	Saudi Arabia	9342	123	26.9
5	Pakistan	1364	29	47.0
6	Turkey	3465	84	42.3
7	Iraq	7289	200	69.0
8	Philippines	2452	42	37.8
9	Indonesia	821	33	59.1
10	Israel	17,643	124	57.2
11	Qatar	43,441	74	45.3
12	Kazakhstan	5680	87	27.0
13	Kuwait	22,289	131	34.5
14	Oman	17,579	154	36.8
15	China	59	3	48.5
16	UAE	8096	40	52.3
17	Japan	599	11	36.5
18	Singapore	9811	5	38.0
19	Bahrain	35,589	124	22.9
20	Nepal	1892	12	47.4
21	Afghanistan	993	36	55.0
22	Palestine	6119	44	27.8
23	S. Korea	437	7	43.0
24	Lebanon	3645	36	42.0
25	Malaysia	306	4	58.4
26	Thailand	50	0.8	56.6
27	Syria	203	9	36.2
28	Sri Lanka	152	0.6	48.3
29	Jordan	345	3	51.5
30	Georgia	642	5	31.8
31	Cyprus	1269	18	62.7
32	Vietnam	11	0.4	58.2
33	Taiwan	21	0.3	72.3
34	Brunei	331	7	50.4
35	Australia	1046	32	63.0
36	Russia	7358	129	43.7
37	New Zealand	360	5	52.0

### Statistical Analysis

Descriptive statistics were reported in the form of frequency, percentages, mean and standard deviation. As the data was not following the normal distribution, Spearman’s correlation was used to evaluate the relationship between the number of cases per million of populations and deaths per million of the population due to COVID-19 with vitamin D level. Statistical analysis was done with the help of Statistical Package for Social Science (SPSS) version 21.

## Results

Total cases per million and deaths per million for each country and the corresponding mean vitamin D levels have been collated (Table 1). Maximum cases per million and death per million were reported in Qatar and Iran respectively while minimum cases per million and death per million were reported from Vietnam and Taiwan respectively. The highest and lowest concentration of vitamin D was reported from Taiwan and Bahrain respectively.

**Fig. 1:**
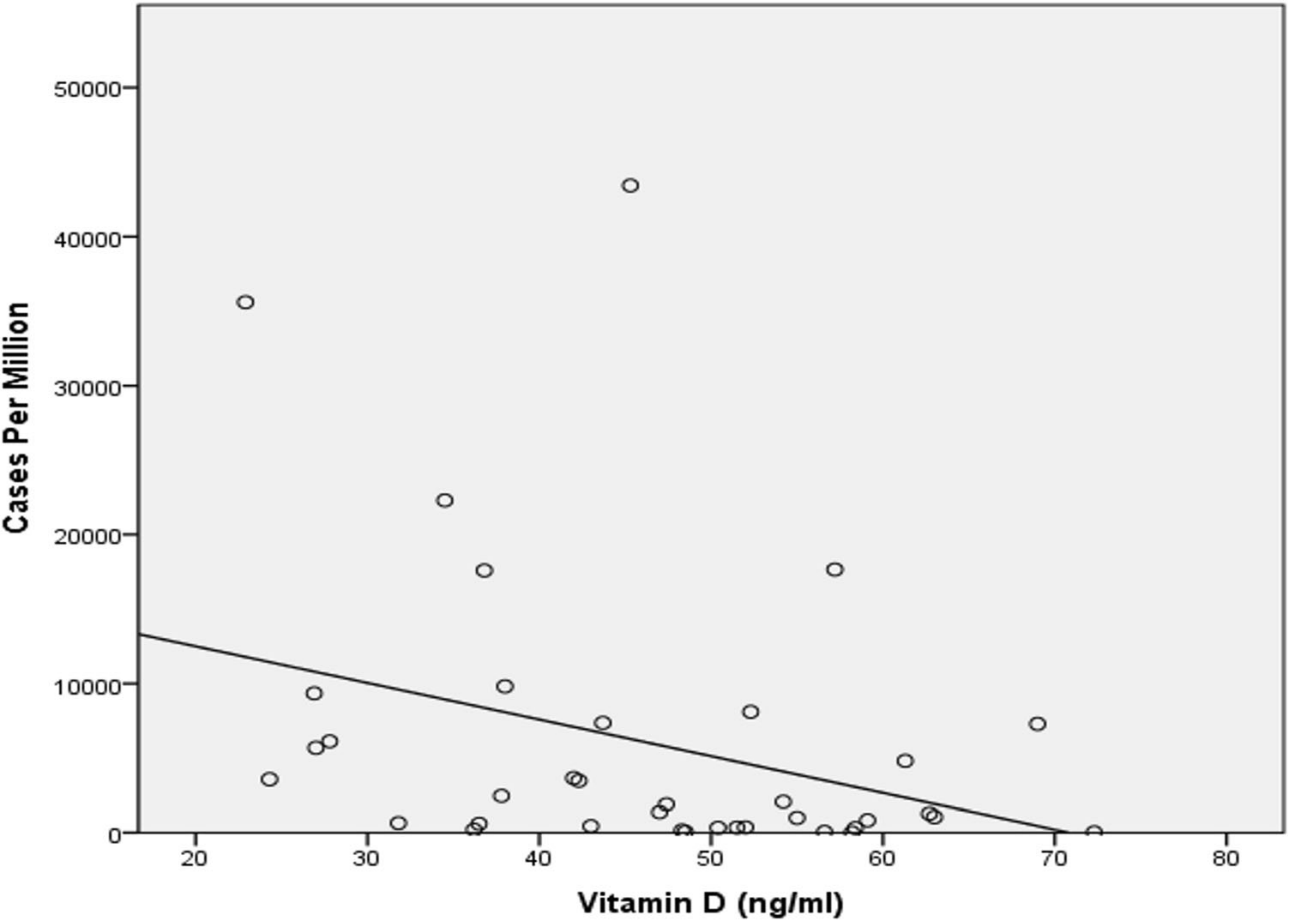
Scatterplot depicting COVID 19 cases per million and Vitamin D levels from different countries

**Fig. 2:**
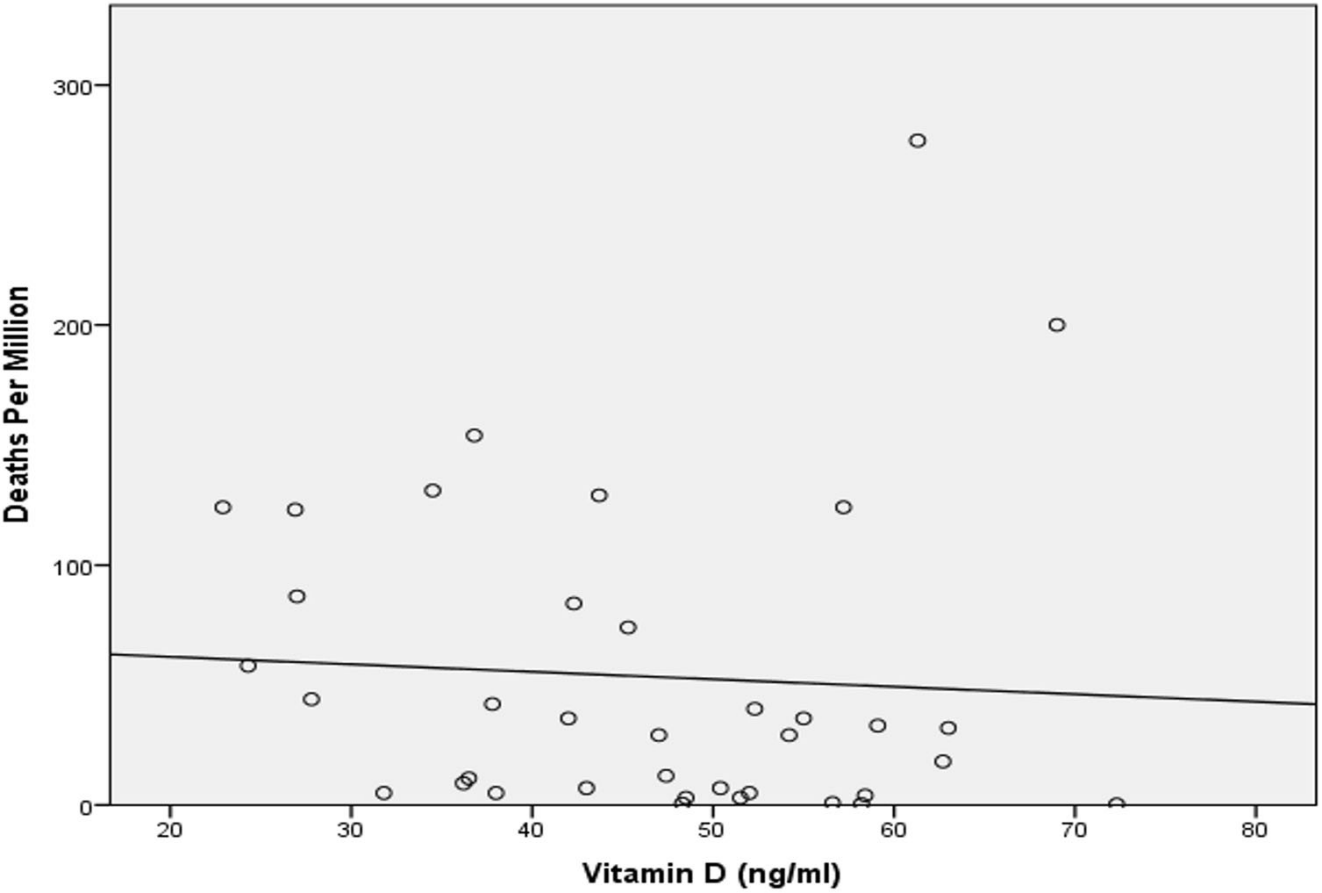
Scatterplot depicting COVID 19 deaths per million and Vitamin D levels from different countries

Negative moderate and statistically significant correlation was observed between cases per million and vitamin D level (r =  − 0.394, *p* = 0.016) [Fig. 1]. Mild negative correlation was observed for death per million and vitamin D level (r =  − 0.280) but this was not statistically significant (*p* = 0.093) [Fig. 2].

## Discussion

In this study, we did the correlative investigation of vitamin D and its effect on mortality of COVID-19 cases in the Asia pacific population. We performed a correlation between Vitamin D levels and the number of cases and deaths per million till 15th September 2020. Vitamin D levels inversely correlated with the number of COVID-19 cases (r =  − 0.394, *p* value = 0.016). The highest concentration of vitamin D as well as the lowest number of deaths per million were reported from Taiwan.Vitamin D regulates the immune system and helps in decreasing the severity of viral infection, mainly related to respiratory systems [14–16]. The active form of Vitamin D 1,25-dihydroxyvitamin D, on binding to vitamin D receptor (VDR), gets translocated into the cell nucleus and attaches with retinoid X receptor (RXR). The RXR-VDR complex binds to Vitamin D Response Elements (VDRE) located in the promoter regions of genes affecting the expression of various genes [17].

Covid 19 causes acute respiratory tract infections, and these infections are more prone in individuals with lower vitamin D levels. Various mechanisms had been postulated to explain the role of Vitamin D in the COVID19 pandemic. Vitamin D is known to modulate RAS activity and angiotensin-converting enzyme-2 (ACE2) expression [18]. SARS-CoV-2, on infecting a host cell, would lead to downregulation of ACE2 expression [19]. ACE2 downregulation is accompanied by exacerbated inflammatory reaction culminating in cytokine storm and lethal ARDS [19]. Animal models have demonstrated an increase in the mRNA and protein levels of ACE2 on vitamin D administration. This subsequently would lead to a decrease in oxygen saturation. Interestingly, Vitamin D upregulates ACE 2 expression and mitigate inflammatory responses by its anti-inflammatory properties. An increased prevalence of Vitamin D deficiency in many countries can lead to inefficient mitigation of inflammatory responses by increasing ACE 2, leading to increased mortality in COVID patients [12]. A cell culture study done on Vero E6 cell lines demonstrated that even after the entry of SARS- COV 2 into the cells, Vitamin D was able to have an inhibitory effect on the replication of the virus [20]. Animal model studies have attributed this inhibitory effect of vitamin D action on ACE 2 [21]. Aartjan J.et al. [22] showed that in cell culture, zinc inhibits RNA-dependent RNA polymerase. SARS- COV2 being an RNA virus, zinc can inhibit replication of these viruses. Vitamin D deficiency had been associated with a decrease in serum zinc levels. So vitamin D supplementation would be beneficial in such patients to normalize the serum zinc levels [23]. A combination supplement of Vitamin D and zinc in COVID- 19 patients had shown a decreased incidence of cytokine storm by its immunomodulating activity [24].

Further, Vitamin D supplemented mice demonstrated milder symptoms of ARDS [25]. Vitamin D also reduces the generation of pro-inflammatory cytokines and increases the expression of anti-inflammatory cytokines, thereby mitigating the development of severe lung damages due to cytokine storm [26, 27]. Apart from the above-mentioned mechanisms, Vitamin D also contributes to the maintenance of the integrity of the physical barrier via E-cadherin [28].

A similar study was conducted by Ilie et al. (2020) for 20 European countries and concluded that the population with Vitamin D deficiency are most vulnerable for COVID-19 [29]. Respiratory tract infections decreased as vitamin D concentration increased between 20–30 ng/mL [14]. To achieve these levels, 2000–5000 IU/d of vitamin D should be administered to all individuals with lower vitamin D levels [30]. Loading doses are required to achieve levels between 40–60 ng/mL. Also, vitamin D should be administered to all individuals with severe vitamin D deficiency to decrease the severity of COVID-19. Further, in the Asia–pacific region where people have adequate sunlight exposure, vitamin D deficient patients should be advised to have a healthy lifestyle with appropriate sun exposure in addition to the vitamin D rich diet. However, vitamin D supplementation should be administered with caution and should not be taken without a medical prescription to prevent hypervitaminosis D manifesting as fatigue, irritability, nausea, vomiting, dizziness, confusion and nervousness, hypercalcemia, and hypercalciuria [31].

Although, various potential mechanisms have been implicated in the possible use of Vitamin D in mitigating COVID-19 progression including regulation of RAS network, changes in the transcriptional activity of Vitamin D receptor, expression of antimicrobial peptides, and regulation of inflammatory response; clinical trials are required to substantiate the claims [32]. But, due to multiple confounders like dexamethasone in hospital-based treatment trials, the evidence for the effect of Vitamin D in COVID-19 will have to be assessed by population-based trials indicating the prophylactic efficiency of Vitamin D [33]. The inverse correlation of Vitamin D and COVID-19 cases observed in our study also brings into focus the need to investigate other compounds that increase the VDR expression and have actions similar to Vitamin D. Quercetin is a nonsteroidal natural compound that activates VDR and its downstream pathways [34]. Similarly, Dexamethasone has been shown to increase VDR levels and accentuate downstream signaling [35]. The use of these drugs, alone or in combination with Vitamin D, has been hypothesized to be therapeutically beneficial in COVID-19 due to their immune-modulatory effect and regulation of expression of genes [36, 37]. Further, the ability of vitamin D to supplement the immune modulatory action of Hydroxychloroquine is being explored in clinical trials [38, 39].

### Limitations

The study hasn’t considered other country-wise confounding factors that can lead to a difference in the number of COVID-19 cases in different countries. It also important to note that correlation does not prove causation, this just gives us an indication for a hypothesis. The role of vitamin D needs to be explored in COVID 19 through clinical trials.

## Conclusion

The negative correlation observed between Vitamin D levels and the number of COVID-19 cases in the Asia Pacific region points to the possible protective effect of Vitamin D in blunting the infectivity of COVID-19 in causing debilitating infection in the countries in the Asia Pacific region. The multitude of factors that affects the COVID-19 infectivity and mortality calls for adequately designed population-based prospective studies to understand the prophylactic efficiency of Vitamin D and other analogs that modulate the VDR signaling. The emergence of evidence from such studies would help in defining policies regarding prophylactic supplementation of Vitamin D.
